# Concurrent manifestation of oligodontia and thrombocytopenia caused by a contiguous gene deletion in 12p13.2: A three‐generation clinical report

**DOI:** 10.1002/mgg3.679

**Published:** 2019-04-04

**Authors:** Jamila Ross, Willem Fennis, Nicole de Leeuw, Marco Cune, Annemieke Willemze, Antoine Rosenberg, Hans‐Kristian Ploos van Amstel, Marijn Créton, Marie‐José van den Boogaard

**Affiliations:** ^1^ Department of Oral‐Maxillofacial Surgery, Prosthodontics and Special Dental Care University Medical Center Utrecht Utrecht the Netherlands; ^2^ Department of Human Genetics Radboud University Medical Center Nijmegen the Netherlands; ^3^ Center for Dentistry and Oral Hygiene, Department of Fixed and Removable Prosthodontics and Biomaterials University Medical Center Groningen Groningen the Netherlands; ^4^ Department of Oral‐Maxillofacial Surgery, Prosthodontics and Special Dental Care St. Antonius Hospital Nieuwegein Nieuwegein the Netherlands; ^5^ Department of Hematology University Medical Center Utrecht Utrecht the Netherlands; ^6^ Department of Genetics University Medical Center Utrecht Utrecht the Netherlands

**Keywords:** contiguous gene deletion, *ETV6*, *LRP6*, oligodontia, thrombocytopenia

## Abstract

**Background:**

Wnt and Wnt‐associated pathways play an important role in the genetic etiology of oligodontia, a severe form of tooth agenesis. Loss‐of‐function mutations in LRP6 , encoding a transmembrane cell‐surface protein that functions as a coreceptor in the canonical Wnt/b‐catenin signaling cascade, also contribute to genetic oligodontia.

**Methods and results:**

We describe a three‐generation family with hereditary thrombocytopenia and oligodontia. Genome wide array analysis was performed. The array results from the index patient revealed an interstitial loss of 150 kb in 8p23.1 (chr8:6,270,299–6,422,558; hg19) encompassing *MCPH1* and *ANGPT2* and an interstitial loss of 290 kb in 12p13.2 (chr12:12,005,720–12,295,290; hg19) encompassing *ETV6*, *BCL2L14* and *LRP6*.

**Conclusion:**

This case report shows a three‐generation family with hereditary thrombocytopenia and oligodontia with a heterozygous 290 kb novel contiguous gene deletion in band p13.2 of chromosome 12, encompassing *LRP6* and *ETV6*. In this report we discuss the clinical relevance of the deletion of both genes and illustrate the importance of thorough examination of oligodontia patients. Comprising not only the oral status but also the medical history of the patients and their relatives.

## 1 INTRODUCTION

Tooth agenesis or hypodontia is a developmental anomaly, in which one or more permanent teeth fail to develop. It has a reported prevalence of 5.5% in Europe (Polder, Van't Hof, Van der Linden, Frans PGM, & Kuijpers‐Jagtman, 2004). Common forms affecting one or a few absent teeth represent the great majority of cases. Severe hypodontia or oligodontia is defined as the absence of six or more teeth excluding the third molars. These types are estimated to be present in 0.14% of the Caucasian population, with a higher incidence in women than in men (Polder et al., 2004). Congenital absence of teeth is seen as an isolated trait or as part of a syndrome in case of concurring nondental anomalies, and is caused by both (epi‐) genetic as well as environmental factors (van der Weide, Yvonne Schalk, Prahl‐Andersen, & Bosman, 1993).

Over the last few decades, an increasing number of genes involved in embryo‐ and tooth‐ development have been associated with non‐syndromic tooth agenesis (Yu, Wong, Han, & Cai, 2018). Most causal genes encode for components of three interacting signaling pathways (Wnt/β‐catenin, the TGF‐β/BMP and the Eda/Edar/NF‐κB pathways) contributing to a complex development signaling network orchestrating tooth morphogenesis. Mutations in only seven genes (i.e. *AXIN2* [MIM: 604,025], *EDA* [MIM: 300,451], *MSX1* [MIM: 142,983], *PAX9* [MIM: 167,416], *WNT10A* [MIM: 606,268], *WNT10B* [MIM: 601,906], and *LRP6* [MIM: 603,507]) are responsible for the majority of cases with non‐syndromic tooth agenesis (Yu et al., 2018).

*LRP6* (low density lipoprotein receptor related protein 6) encodes a transmembrane cell‐surface protein. It functions as a WNT coreceptor with members from the Frizzled protein family in the canonical Wnt/β‐catenin signaling cascade. Heterozygous loss‐of‐function mutations in *LRP6* were found to cause oligodontia in three patients (Massink et al., 2015).

*ETV6* (MIM: 600,618) encodes an ETS family transcription factor, which binds DNA via a highly conserved C‐terminal DNA‐binding domain. *ETV6* is known to be of interest in hematopoiesis and embryonic development and of major importance in regulating megakaryocytes and platelets (Hock & Shimamura, 2017) Zang et al reported on three families with dominantly inherited thrombocytopenia and a predisposition for hematological malignancies caused by heterozygous germline *ETV6* mutations (Zhang et al., 2015). Additional studies confirm these findings (Duployez et al., 2018; Melazzini et al., 2016; Moriyama et al., 2015; Noetzli et al., 2015; Paulsson et al., 2010; Poggi et al., 2017; Topka et al., 2015). To date, more than 80 germline *ETV6* mutation carriers from 22 families and one pedigree with an intragenic deletion of *ETV6* are reported. (Duployez et al., 2018; Paulsson et al., 2010) Nevertheless, the exact understanding of the clinical impact of *ETV6* mutations and the physiological role of *ETV6* remains to be elucidated. Most individual germline *ETV6* mutations have been identified in single families or patients. It is yet unclear whether different mutations result in different predispositions to develop specific types of malignancy or are associated with different risks of bleeding (Hock & Shimamura, 2017).

In this clinical report we describe two boys, their father and their paternal grandmother with resembling dental phenotypes caused by a 290 kb deletion in band p13.2 of chromosome 12. This deletion contains *LRP6* but also *ETV6*, which could explain the characteristic phenotype described.

## 2 CLINICAL PRESENTATION

**Figure 1:**
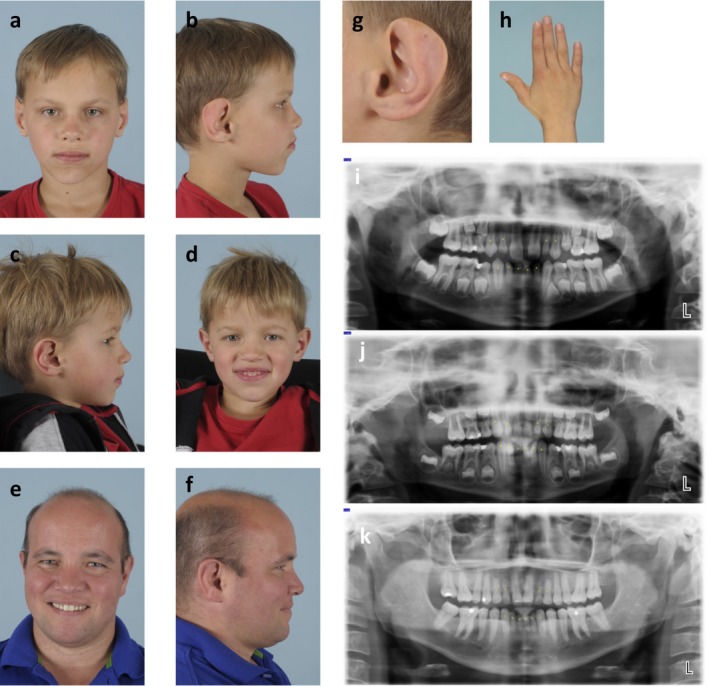
(a) Index patient (proband III‐5). (b) Index patient (proband III‐5). (c) Proband III‐6. (d) Proband III‐6. (e) Proband II‐3. (f) Proband II‐3. (g) Left ear of the index patient, note the mostly absent helix. (h) Slight underdevelopment of the thumb of the index patient. (i) Dental panoramic radiograph of the index patient. Yellow dots indicate deciduous teeth. (j) Dental panoramic radiograph proband III‐6. Yellow dots indicate deciduous teeth. (k) Dental panoramic radiograph proband II‐3. Yellow dots indicate deciduous teeth

The index patient (proband III‐5, Figure 1a,b) was a 10‐year old boy born at 39 + 6 weeks after a troubled pregnancy with regular cardiotocogram abnormalities. At birth his weight was 3,210 g. There were no major deviations in growth and (cognitive) development. On physical examination he had sparse hair, mild sparseness of the eyebrows, agenesis of various permanent teeth, taurodontia, dysmorphic ears with an under folded helix, and slight underdevelopment of the distal phalanx of the thumbs (Figure 1g,h).

**Figure 2:**
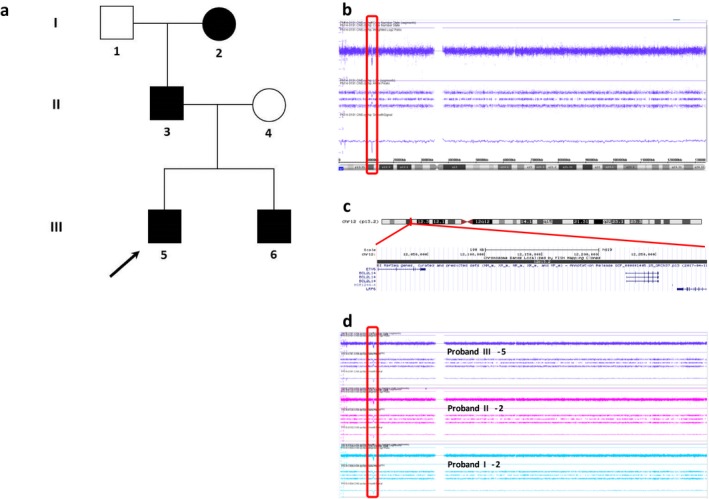
(a) A three‐generation‐pedigree of familial hypodontia and thrombocytopenia with the affected family members indicated by solid black squares (males) or circles (females). The index patient (proband III‐5) is indicated by an arrow. (b) Array plot of chromosome 12 of the index patient with an interstitial deletion of 290 kb in 12p13.2 (red rectangle). (c) Schematic representation of chromosome 12 with the p13.2 region enlarged in the lower part of the figure, showing a screen shot of the UCSC Genome Browser Build 37/hg19 (http://genome.ucsc.edu/). The genes located in the deleted 12p13.2 region are shown below the chromosome bands. (d) Array plot of chromosome 12 of the index patient (proband III‐5), his father (proband II‐3) and the paternal grandmother (proband I‐2), each showing an interstitial deletion of 290 kb in 12p13.2 (red rectangle).

Together with his 37‐year old father (proband II‐3, Figure 1e,f), his younger brother (proband III‐6: six years old, Figure 1c,d) and his paternal grandmother (proband I‐2)he consulted a clinical geneticist for a suspicion of hereditary thrombocytopenia. Figure 2a shows the corresponding three‐generation‐pedigree. In addition to the increased bleeding tendency, the index patient, his younger brother and their father had an almost identical tooth agenesis pattern. They all lack the following permanent teeth: 1.2, 1.3, 2.2, 2.3, 3.1, 3.2, 4.1, 4.2 and 4.3. Additionally, the father and his youngest son lack the lower left permanent canine (3.3). Figure 1i‐k show the dental panoramic radiographs. Corresponding Tooth Agenesis Codes (TAC) are 006.006.007.007 (probands II‐3 and III‐6) and 006.006.003.007 (proband III‐5). The TAC is a method based on the binary number system, used to describe patterns of missing teeth. Depending on the number and location of missing teeth, every possible pattern of tooth agenesis has a unique TAC (Van Wijk & Tan, 2006); (Creton, Cune, Verhoeven, & Meijer, 2007). Questions on family history revealed similar dental abnormalities in the paternal grandmother (proband I‐2), however she currently wears a full dental prosthesis and no documentation is available to confirm an oligodontia diagnosis.

## 3 MICRO ARRAY ANALYSIS

DNA was extracted from peripheral blood using standard procedures. Genome wide array analysis was performed on DNA using the Affymetrix CytoScan HD array platform (Affymetrix, Inc., Santa Clara, CA, USA) following the manufacturer's protocols. The array results from the index patient (proband III‐5) revealed an interstitial loss of 150 kb in 8p23.1 (chr8:6,270,299–6,422,558; hg19) encompassing *MCPH1* (MIM: 607,117, NM_024596.5) and *ANGPT2* (MIM: 601,922, NM_001147.2) and an interstitial loss of 290 kb in 12p13.2 (chr12:12,005,720–12,295,290; hg19) encompassing *ETV6* (NM_001987.4), *BCL2L14* (MIM: 606,126, NM_030766.1) and *LRP6* (NM_002336.3). (according to ISCN 2016 nomenclature the genotype of the index patient is: arr[GRCh37] 8p23.1(6270299_6422558)x1,12p13.2(12005720_12295290)x1).

With subsequent carrier testing by array in the parents, both losses were also detected in the similarly affected father. Next, the same loss in 12p13.2 was also detected by array in proband III‐6 and the brothers’ paternal grandmother (proband I‐2), who were both similarly affected (Figure 2b‐d). They did not have a loss in 8p23.1.

## 4 DISCUSSION

We describe a three‐generation family affected by a deletion in 12p13.2. This deletion encompasses not only part of*LRP6* , but also *BCL2L14* and part of*ETV6* .*ETV6* plays an important role in hematopoiesis and is established as a major intrinsic regulator of megakaryocytes and platelets (Hock & Shimamura, 2017). Individuals with a mutation in this gene have an increased susceptibility to thrombocytopenia, hematologic malignancies and possibly solid neoplasms. All, except one, of the germline *ETV6* mutations, cluster within the highly conserved ETS domain responsible for binding to DNA (Hock & Shimamura, 2017). To our knowledge, there is only one pedigree reported with a deletion comprising exon 2 (including the PNT domain) (Paulsson et al., 2010). The deletion found in our proband and affected family members encompasses the last five exons (4, 5, 6, 7, 8) of*ETV6* which includes the ETS domain (Exon 6, 7, 8). This is thus likely to explain their reported chronic thrombocytopenia and increased bleeding tendency. The family history does not reveal hematologic malignancies or solid neoplasms.

How germline *ETV6* mutations predispose to malignancy remains poorly understood. Although, *ETV6* has been identified as a fusion partner in different chromosomal translocation oncogenes and somatic *ETV6‐RUNX1* fusions are common in childhood ALL, a somatic *ETV6‐RUNX1* fusion is reported in just one case with a germline *ETV6* variant (Moriyama et al., 2015). In our family a fusion of *ETV6* and *LRP6* is not expected, while the transcription of both genes are in opposite direction. However, the well‐defined association of *ETV6* germline mutations and hematologic malignancies, would entail a predisposition to malignancies in this family.

The deletion of the last eight exons (exon 16–23) of*LRP6* explains the extensive agenesis of teeth (3). This is the first deletion reported for*LRP6* as a cause of hypodontia. Noteworthy is the almost identical pattern of tooth agenesis shown in the father and his two sons. Generally, the presentation of the dentition in oligodontia is heterogeneous, with highly variable numbers and patterns of missing teeth between affected family members (Creton et al., 2007; Dreesen, Swinnen, Devriendt, & Carels, 2013). The strikingly similar pattern of tooth agenesis in this family seems to indicate an important role for genetics in determining the pattern of tooth agenesis.

The Database of Genomic Variants (DGV), which contains genomic variations observed in healthy individuals (http://genome.ucsc.edu and http://projects.tcag.ca/variation/) reports one additional contiguous deletion of *LRP6* and *ETV6*, several separate small deletions of *LRP6* and *ETV6* and one extended deletion of *LRP6*. One might hypothesize that in these healthy individuals the associated tooth agenesis and thrombocytopenia are not identified as congenital malformations or the features are subclinical.

Previous reported families with tooth agenesis caused by a *LRP6* mutation appeared to be nonsyndromic. Nevertheless, both Massink and Ockeloen independently reported cases showing minor anatomical variations of the ear and underdevelopment of the fingers or thumb (Massink et al., 2015; Ockeloen et al., 2016). In addition to a number of other subtle physical features our proband also displays these minor anatomical variations: dysmorphic ears with a severe under folded helix and slight underdevelopment of the distal phalanx of the thumbs. Paying specific attention to these characteristics, we noticed these minor dysmorphic features also in other patients carrying a *LRP6* mutation. This might implicate that the ear and thumb could serve as a detection marker for a deletion or mutation involving*LRP6* . Further research is necessary to support this association.

Apart from parts of*LRP6* and the *ETV6* , the deletion also encompasses*BCL2L14* . The corresponding protein belongs to the BCL2 family of proteins, which comprises both pro‐ and antiapoptotic regulators of programmed cell death. To our knowledge, so far no disorders have been associated to *BCL2L14* mutations or deletions.

In summary, this case report describes a family with oligodontia and thrombocytopenia due to a deletion encompassing both*LRP6* and*ETV6* illustrating the importance of examining apparent isolated hypodontia patients more extensively. Besides dental examination, thorough consultation should consist of extensive questioning on the medical history of the patient and his or her relatives. Hence, seeing these young patients with tooth agenesis in a multidisciplinary setting and/or referring for genetic counseling can aid in the early recognition of associated disorders. Seen in this light, genetic counseling of individuals with oligodontia should thereby not solely consist of mutation identification in possible causative genes, but also a contiguous gene deletion should be considered.

## CONFLICT OF INTEREST

All authors declare that they do not have any conflict of interest.
